# Deep Vein Thrombosis in the Uninjured Limb in Patients with Lower Extremity Fractures: A Retrospective Study

**DOI:** 10.1155/2020/1647617

**Published:** 2020-06-22

**Authors:** Peng-Fei Wang, Jia-Hao Li, Chen Fei, Zhi Li, Chao Ke, Kun Shang, Yu-Xuan Cong, Shuang-Wei Qu, Bin-Fei Zhang, Yan Zhuang, Kun Zhang

**Affiliations:** ^1^Department of Orthopedic Trauma, Honghui Hospital, Xi'an Jiaotong University, Xi'an, Shaanxi Province 710054, China; ^2^Department of Hand and Foot Microsurgery, Baoji Hospital of Traditional Chinese Medicine, Baoji, Shaanxi Province 721000, China

## Abstract

**Objective:**

This study is aimed at investigating the incidence of deep vein thrombosis (DVT) in the uninjured limb during hospitalization and 1 month after surgery in patients with lower extremity fractures.

**Methods:**

We collected the clinical data of patients with lower extremity fractures in Xi'an Honghui Hospital. Doppler ultrasonography was used to diagnose DVT. According to the results of ultrasonography, the patients were divided into two groups: uninjured limb with DVT group and uninjured limb without DVT group.

**Results:**

A total of 494 patients who met all inclusion criteria were included in this study. The incidence rate of DVT in the uninjured limb was 19.84% and 18.83% during hospitalization and 1 month after surgery, respectively. Age (OR = 1.035, 95% CI: 1.013–1.059; *P* = 0.002) and D-dimer level 1 day after surgery (OR = 1.065, 95% CI: 1.030–1.102; *P* < 0.001) were independent risk factors for DVT during hospitalization. Similarly, age (OR = 1.045, 95% CI: 1.021–1.070; *P* < 0.001) and D-dimer level 1 day after surgery (OR = 1.048, 95% CI: 1.014–1.083; *P* = 0.006) were independent risk factors for DVT 1 month after surgery. During hospitalization and 1 month after surgery, 15.79% and 12.35% of patients had double lower limb thrombosis and 4.04% and 6.48% of patients had DVT in the uninjured limb only, respectively.

**Conclusion:**

The actual incidence of DVT in the uninjured limb in patients with lower extremity fractures cannot be ignored despite the use of anticoagulants for prevention or treatment during hospitalization. We should also be aware of DVT in the uninjured limb while focusing on DVT in the injured limb.

## 1. Introduction

Deep vein thrombosis (DVT) is a potentially life-threatening complication that is common in trauma patients and that can occur early during hospitalization. Therefore, the selection of patients at risk for DVT and early initiation of prophylaxis are important factors for improving outcome [1]. The incidence of DVT is significantly increased in patients with lower extremity fractures because of risk factors such as staying in bed for a long time, surgery, and trauma. Therefore, screening and preventing DVT during hospitalization have received extensive attention [2].

Previous studies have shown that in the absence of preventive measures for venous thromboembolism (VTE), the incidence rate of postoperative VTE can range from 40% to 60%, and the incidence of DVT and pulmonary embolism (PE) is higher and lower, respectively, in China than in other countries [3–5]. All these data come from observing injured limbs. However, the uninjured limb could also be at risk for DVT. Studies have shown that DVT can occur not only in the injured limb but also in the uninjured limb [6, 7]. Decker and Weaver showed that DVT could occur in both the injured and uninjured legs with a trend toward a higher incidence in the injured leg [8]. Mok et al. reported that the incidence rate of DVT in the uninjured limb is 14.3% [9].

According to the classic theory of Virchow, venous wall injury, slow blood flow, and hypercoagulability are the three factors that cause DVT [10]. Kaperonis et al. found that 5-day bed rest in a normal person results in sluggish blood flow, increased aggregation of red blood cells, and increased blood viscosity [11]. Furthermore, because of long-term fixation and traction in patients with lower extremity fractures, their physical activity was reduced and the bedtime was prolonged, leading to muscle atrophy and blood coagulation, which could induce DVT.

Anticoagulant prophylaxis is currently considered one of the most effective methods to decrease the risk of lower extremity DVT [12]. The American College of Chest Physicians Evidence-Based Clinical Practice Guidelines recommend that patients undergoing major orthopaedic surgery should be administered low-molecular-weight heparin (LMWH) to prevent lower extremity DVT [13]. Although DVT has gained increased attention from clinicians, DVT in the uninjured limb in patients with lower extremity fractures is often overlooked.

Thus, the main purpose of this study was to investigate DVT in the uninjured limb in patients with lower extremity fractures during hospitalization and 1 month after surgery.

## 2. Materials and Methods

This study retrospectively analysed the data of patients with lower extremity fractures between September 1, 2014, and October 1, 2017, in Xi'an Honghui Hospital. The study was approved by the ethical board of Honghui Hospital, Xi'an Jiaotong University (No. 2014026).

### 2.1. Inclusion and Exclusion Criteria

The inclusion criteria were as follows: (a) age ≥ 16 years, (b) fresh lower extremity fractures (within three weeks of injury) requiring surgical treatment, and (c) availability of results of in-hospital and 1-month postoperative ultrasonography.

The exclusion criteria were as follows: (a) delayed lower extremity fractures, (b) open soft tissue fractures, and (c) poor compliance.

### 2.2. Treatment

All patients admitted to the hospital were routinely assessed for thromboembolism risk using the risk assessment profile for thromboembolism score [14]. For patients without contraindications to anticoagulation, LMWH (3800 IU/0.4 mL, once per day; Fraxiparine, Glaxo Wellcome Production, GlaxoSmithKline) was subcutaneously injected to prevent DVT. Moreover, mechanical thromboprophylaxis (pressure pump, 20 min, twice per day) was used.

We used Doppler ultrasonography to diagnose DVT. The diagnostic criteria were according to the criteria of Dauzat et al. [15]. The sonographers are blinded to the D-dimer results and results of previous ultrasonography examinations, avoiding the performing bias. Patients were examined during hospitalization and 1 month after surgery. All patients underwent double lower limb ultrasonography. DVTs were classified into three types: central (femoral and iliac veins), peripheral (calf muscle, fibular, and anterior/posterior tibial veins), and mixed thromboses (both central and peripheral thromboses).

According to the results of ultrasonography, the patients were divided into two groups: uninjured limb with DVT group and uninjured limb without DVT group. For patients without DVT, LMWH (3800 IU/0.4 mL, once per day; Fraxiparine, Glaxo Wellcome Production, GlaxoSmithKline) was administered via continuous subcutaneous injection to prevent DVT. For patients with DVT, LMWH (3800 IU/0.4 mL, twice per day; Fraxiparine, Glaxo Wellcome Production, GlaxoSmithKline) was subcutaneously injected to treat DVT. When central or mixed DVT was detected on ultrasonography, an inferior vena cava filter was used to prevent fatal pulmonary embolism, followed by orthopaedic surgery. Anticoagulant therapy was stopped 12 h before surgery and resumed 24 h after surgery. Upon patient discharge, the protocol was changed to rivaroxaban (prophylactic dose: 10 mg/time^/^day; therapeutic dose: 20 mg/time^/^day) until 35 days after surgery.

### 2.3. Statistical Analysis

Statistical analysis was performed using SPSS Version 19.0 (SPSS Inc., Chicago, Illinois, USA). The measurement data were determined if they were normally distributed. An independent sample *t*-test was used for statistical analysis. For enumeration data, a chi-square test was used. Risk factors showing a significant difference in the univariate analysis were further assessed using multivariate logistic regression analysis. *P* values less than 0.05 were considered significant.

## 3. Results

### 3.1. General Information

Using the abovementioned criteria, 494 patients with surgically treated lower extremity fractures were included in this study. There were 228 (46.2%) males and 266 (53.8%) females, with an average age of 58.91 ± 18.88 years (16–94 years). The fractures were located above the knee joint in 290 patients (58.7%), around the knee joint in 101 patients (20.4%), and below the knee joint in 103 patients (20.9%). All patients underwent surgery. The average time interval between fracture occurrence and surgery and between surgery and discharge was 5.78 ± 3.71 days and 5.20 ± 2.50 days, respectively. No patient had pulmonary embolism and abnormal bleeding events associated with lower extremity DVT.

### 3.2. DVT during Hospitalization

Ultrasonography results showed that 98 patients (19.84%) had DVT in the uninjured limb, whereas 396 patients (80.16%) did not have DVT in the uninjured limb. There were 90 (18.2%) with peripheral DVT, 1 (0.2%) with central DVT, and 7 (1.4%) with mixed DVT. The majority of DVTs were of the type, which accounted for 91.8% (90/98) of all DVTs. It is worth mentioning that of the 98 patients with DVT in the uninjured limb, 74 (75.51%) had DVT in both lower limbs and 24 (24.49%) had DVT in the uninjured limb only.

### 3.3. Univariate and Multivariate Analyses of DVT during Hospitalization

In the univariate analysis (Table 1), age (*P* < 0.001), gender (*P* = 0.003), types of fracture (*P* < 0.001), hypertension (*P* = 0.005), coronary heart disease (*P* = 0.001), American Society of Anesthesiologists (ASA) classification (*P* = 0.013), D-dimer level 1 day after surgery (*P* < 0.001), and D-dimer level 1 month after surgery (*P* < 0.001) were significantly associated with DVT during hospitalization.

We performed multivariate logistic regression analysis with the presence or absence of DVT as the outcome variable and the abovementioned patient or injury characteristics as the potential predictor variables. The results showed that age (OR = 1.035, 95% CI: 1.013–1.059; *P* = 0.002) and D-dimer level 1 day after surgery (OR = 1.065, 95% CI: 1.030–1.102; *P* < 0.001) were independent risk factors for perioperative DVT (Table 2).

### 3.4. DVT 1 Month after Surgery

All patients underwent ultrasonography 1 month after surgery. Ultrasonography results showed that the incidence rate of DVT in the injured limb was 33%. Moreover, 93 patients (18.83%) had DVT in the uninjured limb, whereas 401 patients (81.17%) did not have DVT. There were 84 (17.00%) with peripheral DVT, 3 (0.6%) with central DVT, and 6 (1.2%) with mixed DVT. The majority of DVTs were of the peripheral type, which accounted for 90.32% (84/93) of all DVTs. It is worth mentioning that of the 93 patients with DVT in the uninjured limb, 61 (65.59%) had DVT in both lower limbs and 32 (34.41%) had DVT in the uninjured limb only.

### 3.5. Univariate and Multivariate Analyses of DVT 1 Month after Surgery

In the univariate analysis (Table 3), age (*P* = 0.001), gender (*P* = 0.012), types of fracture (*P* = 0.003), hypertension (*P* = 0.025), coronary heart disease (*P* = 0.002), ASA classification (*P* = 0.021), D-dimer level 1 day after surgery (*P* = 0.002), and D-dimer level 1 month after surgery (*P* < 0.001) were significantly associated with DVT 1 month after surgery.

Multivariate logistic regression analysis showed that age (OR = 1.045, 95% CI: 1.021–1.070; *P* < 0.001) and D-dimer level 1 day after surgery (OR = 1.048, 95% CI: 1.014–1.083; *P* = 0.006) were independent risk factors for DVT 1 month after surgery (Table 4).

### 3.6. Dynamic Changes in DVT during Hospitalization and 1 Month after Surgery

We investigated the incidence of DVT in the uninjured limb in 494 patients during hospitalization and 1 month after surgery and found that DVT had changed in 81 patients (16.4%) but did not change in 413 patients (83.6%). Among them, 43 patients had DVT in the uninjured limb during hospitalization, and the thrombosis disappeared 1 month after surgery. In 38 patients, no DVT in the uninjured limb was detected during hospitalization, but DVT was found 1 month after surgery. Newly developed central DVT occurred in 1 patient, mixed DVT in 2 patients, and peripheral DVT in 35 patients.

## 4. Discussion

We retrospectively investigated the changes in DVT during hospitalization and 1 month after surgery in patients with uninjured lower limbs. We found that the incidence rate of DVT in the uninjured limb was 19.84% and 18.83% during hospitalization and 1 month after surgery, respectively. Mok et al. reported that the incidence rate of DVT in the uninjured limb is 14.3% [9]. The incidence rate in our study was slightly higher probably because we included subjects with all types of lower extremity fracture; in contrast, Mok et al. only studied proximal femoral fractures.

Moreover, Godat et al. reported that the risk of VTE after acute injury is highest during the first 3 months after injury [16]. The study showed that the thrombosis in the uninjured limb was distributed to the peripheral part of the lower limb during hospitalization and 1 month after surgery, with DVT mainly occurring in the intramuscular vein. The intramuscular vein is the most frequently involved vessel in DVT in patients with fractures, which may be related to the appearance of the soleal vein [17]. Moreover, with trauma, the overall blood flow is slow, the blood is at high coagulation state, and patients with postoperative pain are on the bed rather than performing early activity, increasing venous pressure; all of these can result in DVT in the uninjured limb.

According to the study of Lee et al., gender is a risk factor for DVT, with women being more likely to have DVT in the uninjured limb [18]. It may be related to anatomical factors. However, the exact association is unclear. Different types of fracture have a certain effect on the thrombosis in the uninjured limb. This study showed that the incidence rate of DVT in fractures above, around, and below the knee joint was 25.9%, 10.9%, and 11.7%, respectively, with a statistically significant difference (*P* < 0.05). Previous studies [3, 5, 7, 9] have also reported a higher incidence of fractures above the knee, including pelvic fractures, hip fractures, and femoral fractures; most of these occurred with high-energy injuries, bleeding, increased swelling, and severe damage to the blood vessel lining, which can lead to DVT. Other studies [19–21] have also suggested that common medical conditions can increase the incidence of DVT. In this study, although hypertension and coronary heart disease had an effect on DVT in the uninjured limb, multivariate analysis showed that they were not independent risk factors. Mantilla et al. reported that patients with ASA ≥ 3 had an increased risk of clinically relevant VTE within 30 days after lower extremity joint arthroplasty [22]. Our study included patients with ASA ≤ 3; further, we found that the incidence of DVT in ASA 2 and 3 patients was significantly higher than that in ASA 1 patients (*P* < 0.05).

More importantly, through multivariate analysis, we concluded that age and D-dimer level 1 day after surgery were independent risk factors for DVT in the uninjured limb. The elderly is a well-known risk factor for DVT, which was confirmed in a previous study. Makhdom et al. and Goel et al. found age over 40 years to be a significant risk factor for the development of DVT in patients with lower extremity fractures [23, 24]. The elderly is more prone to fractures or may be immobilized owing to medical conditions; hence, we should be able to evaluate the risk of DVT in older patients. Another point to note is that the D-dimer level 1 day after surgery was significantly increased (*P* < 0.05). Jovanovic et al. reported that the D-dimer level was significantly increased in patients with acute DVT and was the preferred index for screening lower extremity DVT [25]. Therefore, D-dimer level is usually considered a reliable screening tool to rule out DVT, and we believe that it could be used to diagnose early DVT in combination with venous ultrasonography of both lower extremities.

In addition, we found that during hospitalization and 1 month after surgery, 15.79% and 12.35% of patients had double lower limb thrombosis, and 4.04% and 6.48% of patients had DVT in the uninjured limb only, respectively. We also found that in 8.7% of patients with DVT in the uninjured limb during hospitalization, the thrombosis disappeared 1 month after surgery, whereas in 7.6% of patients with no DVT in the uninjured limb during hospitalization, DVT was detected 1 month after surgery. Although most new blood clots in the uninjured limb are peripheral DVTs, there is still the possibility of new blood clots in the uninjured limb after surgery, and we should not ignore it.

The symptoms of lower extremity DVT include leg pain, oedema, pigmentation, and ulceration [26]. However, the diagnosis of VTE is difficult in the postoperative setting because signs such as hypoxaemia, leg pain, and swelling are so common [27], especially in patients with lower extremity fractures. Previous studies on the occurrence of DVT in the injured lower limb have been performed, with few studies focusing on its occurrence in the uninjured limb. To the best of our knowledge, we were the first to investigate the incidence of DVT in the uninjured limb.

Our study has several potential limitations. Firstly, the results may be biased by our single-centre, retrospective study design. Secondly, lower extremity venous ultrasonography was used as the diagnostic standard. Although it was noninvasive and reproducible, its diagnostic accuracy was inferior to that of venography and it also had a certain effect on the results. Therefore, a multicentre prospective study with a large sample size will help explore the role of DVT in the uninjured limb in patients with lower extremity fractures.

## 5. Conclusions

In conclusion, the actual incidence of DVT in the uninjured limb in patients with lower extremity fractures cannot be ignored despite the use of anticoagulants for prevention or treatment during hospitalization. We should also be aware of DVT in the uninjured limb while focusing on DVT in the injured limb.

## Figures and Tables

**Table 1:** Patient characteristics according to ultrasound during hospitalization.

	No thrombosis	Thrombosis	Overall	*P*
Number	396	98	494	
Age	56.26 ± 18.95	69.61 ± 14.32	58.91 ± 18.88	<0.001
Gender				
Female	200	66	266	0.003
Male	196	32	228	
Types of fracture				
Above the knee joint fracture	215	75	290	<0.001
Around the knee joint fracture	90	11	101	
Below the knee joint fracture	91	12	103	
Medical morbidity				
Hypertension (%)	61 (15.40)	27 (27.55)	88	0.005
Diabetes (%)	26 (6.57)	11 (11.22)	37	0.117
Coronary heart disease (%)	72 (18.18)	33 (33.67)	105	0.001
Days between fracture and operation (days)	5.86 ± 3.76	5.47 ± 3.51	5.78 ± 3.71	0.241
Days between operation and discharge (days)	5.05 ± 2.39	5.79 ± 2.86	5.20 ± 2.50	0.761
ASA classification				
1	80	8	88	0.013
2	243	65	308	
3	73	25	98	
Duration of operation (mins)	121.65 ± 98.01	114.23 ± 60.29	120.18 ± 91.76	0.354
Blood loss (mL)	254.66 ± 263.51	287.53 ± 223.22	261.28 ± 256.02	0.470
Liquid transfusion (mL)	1799.52 ± 571.24	1720.41 ± 485.03	1783.79 ± 555.60	0.149
Serum markers				
D-dimer at admission (mg/L)	11.73 ± 18.21	15.64 ± 15.70	12.52 ± 17.79	0.723
D-dimer at preoperation (mg/L)	4.13 ± 4.42	5.02 ± 4.14	4.29 ± 4.36	0.919
D-dimer at one day after operation (mg/L)	6.02 ± 6.50	11.44 ± 9.34	7.07 ± 7.47	<0.001

ASA: American Society of Anesthesiologists.

**Table 2:** Risk factor analysis of DVT during hospitalization determined by multivariate logistic regression.

Group	*β* value	OR	95% CI	*P* value
Age	0.035	1.035	1.013-1.059	0.002
Sex	0.442	1.556	0.889-2.723	0.121
Types of fracture	0.239	1.271	0.822-1.964	0.281
Hypertension	0.328	1.388	0.742-2.594	0.305
Coronary heart disease	-0.060	0.942	0.498-1.784	0.855
ASA	0.074	1.077	0.656-1.768	0.771
D-dimer at one day after operation	0.063	1.065	1.030-1.102	<0.001
D-dimer at one month after operation	0.095	1.100	0.989-1.223	0.079

**Table 3:** Patient characteristics according to one month after operation ultrasound.

	No thrombosis	Thrombosis	Overall	*P*
Number	401	93	494	
Age	56.46 ± 18.86	69.47 ± 14.95	58.91 ± 18.88	0.001
Gender				
Female	205	61	266	0.012
Male	196	32	228	
Types of fracture				
Above the knee joint fracture	221	69	290	0.003
Around the knee joint fracture	88	13	101	
Below the knee joint fracture	92	11	103	
Medical morbidity				
Hypertension (%)	64 (15.96)	24 (25.81)	88	0.025
Diabetes (%)	26 (6.48)	11 (11.83)	37	0.078
Coronary heart disease (%)	74 (18.45)	31 (33.33)	105	0.002
Days between fracture and operation (days)	5.70 ± 3.69	6.13 ± 3.78	5.78 ± 3.71	0.796
Days between operation and discharge (days)	5.10 ± 2.43	5.64 ± 2.77	5.20 ± 2.50	0.902
ASA classification				
1	80	8	88	0.021
2	247	61	308	
3	74	24	98	
Duration of operation (mins)	122.00 ± 97.46	112.31 ± 61.14	120.18 ± 91.76	0.441
Blood loss (mL)	250.96 ± 257.61	306.40 ± 245.30	261.28 ± 256.02	0.971
Liquid transfusion (mL)	1801.77 ± 564.52	1705.43 ± 510.41	1783.79 ± 555.60	0.192
Serum markers				
D-dimer at admission (mg/L)	12.35 ± 18.52	13.22 ± 14.44	12.52 ± 17.79	0.619
D-dimer at preoperation (mg/L)	4.17 ± 4.43	4.81 ± 4.06	4.29 ± 4.36	0.975
D-dimer at one day after operation (mg/L)	6.27 ± 6.71	10.64 ± 9.44	7.07 ± 7.47	0.002
D-dimer at one month after operation (mg/L)	1.51 ± 2.16	2.72 ± 4.25	1.73 ± 2.70	<0.001

ASA: American Society of Anesthesiologists.

**Table 4:** Risk factor analysis of DVT one month after operation determined by multivariate logistic regression.

Group	*β* value	OR	95% CI	*P* value
Age	0.044	1.045	1.021-1.070	<0.001
Sex	0.301	1.351	0.771-2.368	0.293
Types of fracture	0.229	1.257	0.804-1.964	0.316
Hypertension	0.344	1.411	0.753-2.644	0.282
Coronary heart disease	-0.018	0.983	0.518-1.863	0.957
ASA	-0.153	0.858	0.518-1.422	0.553
D-dimer at one day after operation	0.047	1.048	1.014-1.083	0.006
D-dimer at one month after operation	0.046	1.047	0.955-1.149	0.327

## Data Availability

The survey was implemented by Xi'an Honghui Hospital. According to relevant regulations, the data could not be shared.

## Abbreviations

DVT: Deep vein thrombosis
LMWH: Low-molecular-weight heparin
BMI: Body mass index
ASA: American Society of Anesthesiologists
OR: Odds ratio.
